# PIF1 promotes phytochrome-regulated growth under photoperiodic conditions in *Arabidopsis* together with PIF3, PIF4, and PIF5

**DOI:** 10.1093/jxb/ert465

**Published:** 2014-01-13

**Authors:** Judit Soy, Pablo Leivar, Elena Monte

**Affiliations:** Departament de Genètica Molecular, Center for Research in Agricultural Genomics (CRAG) CSIC-IRTA-UAB-UB, Campus Univ. Autònoma de Barcelona, Bellaterra, 08193 Barcelona, Spain

**Keywords:** Arabidopsis, EOD-FRp, growth-related gene expression, hypocotyl elongation, PIF1, short day.

## Abstract

Seedlings growing under diurnal conditions display maximal growth at the end of the night in short-day (SD) photoperiods. Current evidence indicates that this behaviour involves the action of PHYTOCHROME-INTERACTING FACTOR 3 (PIF3) together with PIF4 and PIF5, through direct regulation of growth-related genes at dawn coinciding with a PIF3 accumulation peak generated by phytochrome-imposed oscillations in protein abundance. Here, to assess how alterations in PIF3 levels impact seedling growth, the night-specific accumulation of PIF3 was modulated by releasing SD-grown seedlings into continuous light, or by exposing them to a phytochrome-inactivating end-of-day far-red pulse (EOD-FRp). The data show a strong direct correlation between PIF3 accumulation, PIF3-regulated induction of growth-related genes, and hypocotyl elongation, and suggest that growth promotion in SD conditions involves factors other than PIF3, PIF4, and PIF5. Using a *pif1* mutant, evidence is provided that PIF1 also contributes to inducing hypocotyl elongation during the dark period under diurnal conditions. PIF1 displayed constitutive transcript levels in SD conditions, suggesting that phytochrome-imposed oscillations in PIF1 protein abundance determine its accumulation and action during the night, similar to PIF3 and in contrast to PIF4 and PIF5, which oscillate diurnally due to a combination of circadian clock-regulated transcription and light control of protein accumulation. Furthermore, using single and higher order *pif* mutants, the relative contribution of each member of the PIF quartet to the regulation of morphogenesis and the expression of selected growth marker genes under SD conditions, or under SD conditions supplemented with an EOD-FRp, is defined. Collectively, the data indicate that PIF1, PIF3, PIF4, and PIF5 act together to promote and optimize growth under photoperiodic conditions.

## Introduction

Light is a fundamental environmental cue for plants, and photoreceptors of the phytochrome family (phyA–phyE in *Arabidopsis*) perceive the red (R) (660nm) and far red (FR) (720nm) light of the solar spectrum (Schafer and Nagy, 2006). Phytochromes are synthesized in the cytoplasm in the inactive R-absorbing Pr form, and upon R absortion reversibly convert to the active FR-absorbing Pfr form that is rapidly translocated to the nucleus (Nagatani, 2004). Pr to Pfr photoactivation occurs within seconds after absorption of R photons (Linschitz and Kasche, 1966), whereas Pfr to Pr inactivation takes place in FR-enriched environments (Franklin, 2008) and also in the dark. Pfr to Pr reversion in light-grown seedlings transferred to darkness is slow, with a Pfr half-life of ~60min (Rausenberger *et al.*, 2010). Owing to these properties, phytochromes are able to monitor changes in light quality, quantity, and duration to mediate the adaptation of plant growth and development to changes in ambient light conditions, and regulate processes such as germination, de-etiolation, shade avoidance, or diurnal growth (Franklin and Quail, 2010; Casal, 2013).

The central role of the phytochromes (predominantly phyA and phyB) in growth and development is achieved in large part by regulating the abundance of members of the phytochrome-interacting factor (PIF) family of basic helix–loop–helix (bHLH) transcription factors (Bae and Choi, 2008; Leivar and Quail, 2011). The PIFs (PIF1, PIF3, PIF4, PIF5, PIF6, and PIF7 in *Arabidopsis*) accumulate in the nucleus in the dark, and, upon light exposure, associate photoreversibly and specifically with the Pfr form of the phytochromes. This interaction initiates a cascade of transcriptional changes that allows the implementation of the necessary morphological changes to adapt to the new light environment (Castillon *et al.*, 2007; Jiao *et al.* 2007). For some of the PIF members (PIF1, PIF3, PIF4, and PIF5), light-induced interaction with the Pfr phytochrome triggers their rapid phosphorylation, which in turn induces their ubiquitylation and proteolytic degradation via the proteasome system within minutes (Bauer *et al.*, 2004; Park *et al.*, 2004; Shen *et al.*, 2005; Al-Sady *et al.*, 2006; Oh *et al.*, 2006; Nozue *et al.*, 2007; Shen *et al.*, 2007; Lorrain *et al.*, 2008; Shen *et al.*, 2008), establishing a new lower steady-state level in continuous light of ~10% the amount in the dark for PIF3 (Monte *et al.*, 2004). Exposure to light also induces rapid concomitant phyA degradation (half-life of <2h) and a slower and more modest degradation of phyB, which remains relatively abundant and stable in the light (Sharrock and Clack, 2002; Khanna *et al.*, 2007; Al-Sady *et al.*, 2008). phyB degradation has recently been shown to require PIF3 phosphorylation, which establishes a mutually negative feedback loop between phyB and PIF3 potentially through co-degradation of both proteins (Leivar *et al.*, 2012a; Ni *et al.*, 2013). The distinct light stability properties of phyA and phyB underlie their differential roles in the regulation of PIF abundance: whereas phyA and phyB function mostly redundantly in dark to light transitions, phyB is more important in continuous light and during the first dark hours in light to dark transitions (Al-Sady *et al.*, 2006; Monte *et al.*, 2007; Shen *et al.*, 2008; Leivar *et al.*, 2012a; Soy *et al.*, 2012). Under diurnal conditions where light and dark periods alternate, the progressive decline in phyB Pfr during the night period due to dark reversion allows for the progressive re-accumulation of the PIFs in light-grown seedlings upon exposure to darkness (Monte *et al.*, 2004; Shen *et al.*, 2005; Nozue *et al.*, 2007; Soy *et al.*, 2012). Exposure to FR light-enriched environments, such as vegetational shade, low R/FR ratios, or an end-of-day FR pulse (EOD-FRp), also triggers re-accumulation of the PIFs due to phyB Pfr inactivation (Lorrain *et al.*, 2008; Leivar *et al.*, 2012a, *b*; Casal, 2013).

Hypocotyl elongation is maximal in seedlings grown in continuous darkness. Under diurnal conditions with alternating light/dark cycles, the extent of hypocotyl elongation in *Arabidopsis* seedlings depends on the duration of the dark period in a non-linear fashion (Niwa *et al.*, 2009). In short-day (SD) photoperiods, seedlings display rhythmic growth, with maximal elongation rates at the end of the night (Nozue *et al.*, 2007). Elongation in SD is largely due to the combined actions of PIF3, PIF4, and PIF5, which promote growth specifically at the end of the night (Nozue *et al.*, 2007; Niwa *et al.*, 2009; Soy *et al.*, 2012). Precise regulation of their accumulation and time of action under diurnal conditions has been proposed to involve at least two different mechanisms. For PIF4 and PIF5, a coincidence mechanism has been described that combines regulation of *PIF4* and *PIF5* transcript levels by the circadian clock, superimposed on the control of their protein accumulation by light (Nozue *et al.*, 2007; Nusinow *et al.*, 2011; Yamashino *et al.*, 2013). For PIF3, transcript levels are relatively constant, and oscillations of PIF3 protein abundance are imposed by the action of the phytochromes (Soy *et al.*, 2012). The effects of PIF3, PIF4, and PIF5 on diurnal hypocotyl elongation involve the direct regulation of the growth-related genes *PIL1* (*PHYTOCHROME-INTERACTING FACTOR-3 LIKE 1*), *HFR1* (*LONG HYPOCOTYL IN FAR-RED 1*), and *XTR7* (*XYLOGLUCAN ENDOTRANSGLYCOSYLASE 7*) (Soy *et al.*, 2012), which are up-regulated in conditions where hypocotyl elongation is induced (Salter *et al.*, 2003; Lorrain *et al.*, 2008; Hornitschek *et al.*, 2009; Leivar *et al.*, 2009; Nozue *et al.*, 2011), and the regulation of auxin-related genes that oscillate in phase with hypocotyl growth (Michael *et al.*, 2008; Nozue *et al.*, 2011).

The role of PIF3 as a positive regulator of growth under diurnal conditions has been defined previously, and it has been described how phytochrome-imposed oscillations ensure that PIF3 protein progressively accumulates during the dark period to peak just before dawn, at which time it accelerates growth together with PIF4 and PIF5 (Soy *et al.*, 2012). Despite these advances, a complete understanding of how phytochrome-mediated regulation of PIF abundance under diurnal conditions impacts the expression of growth-related genes and hypocotyl elongation, and whether factors other than PIF3, PIF4, and PIF5 might be involved is still lacking. To address these questions, and based on the current model, here PIF3 protein accumulation has been altered specifically during the night hours in SD conditions by treating seedlings with an EOD-FRp, or by substituting the dark period by a continuous white light treatment. These treatments have allowed PIF3 abundance to be correlated with gene expression and growth, and a new role for PIF1 as a contributing factor to the phytochrome-mediated regulation of growth under diurnal conditions has been unveiled.

## Materials and methods

### Seedling growth and hypocotyl measurements

Wild-type and mutant lines used in these studies were in *Arabidopsis thaliana* Columbia ecotype and described elsewhere, and included *pif1-1* (Huq *et al.*, 2004), *pif3-3* (Monte *et al.*, 2004), *pif4-2* (Leivar *et al.*, 2008a), *pif5-3* (Khanna *et al.*, 2007), *pif1pif3*, *pif3pif4pif5*, and *pif1pif3pif4pif5* (Leivar *et al.*, 2008b), *pif4pif5* and *pif1pif4pif5* (Leivar *et al.*, 2012b), *pif3pif4pif5phyB* (Soy *et al.*, 2012), and *phyB-9* (Reed *et al.*, 1993).

Seeds were sterilized and plated on GM medium without sucrose as previously described (Monte *et al.*, 2003). Seedlings were stratified for 4 d at 4 °C in darkness, and then placed in SD conditions [8h white light (85 μmol m^–2^ s^–1^) + 16h dark] for 2 d at 21 °C. During the third day of growth, seedlings were either kept in SD conditions, transferred to continuous white light conditions (WL), or exposed to a pulse of FR (30 μmol m^–2^ s^–1^) (FRp) for 15min before the dark period.

For hypocotyl measurements, seedlings were arranged horizontally on a plate, photographed using a digital camera (Nikon D80), and measured using NIH Image software (ImageJ, National Institutes of Health). At least 30 seedlings for each line were measured to calculate the mean and standard error (SE).

### Protein extraction and immunoblots

Protein extraction and immunoblots were done as described before in Soy *et al.* (2012). Immunodetection of PIF3 was performed using a rabbit anti-PIF3 polyclonal antibody (Al-Sady *et al.*, 2006), incubated overnight with Hikari solution (Nacalai tesque). Peroxidase-linked anti-rabbit (Amersham) secondary antibody and a SuperSignal West Femto chemiluminescence kit (Pierce) were used for detection using a Las4000 Image (Fujifilm).

### Gene expression analysis

**Fig. 1.**
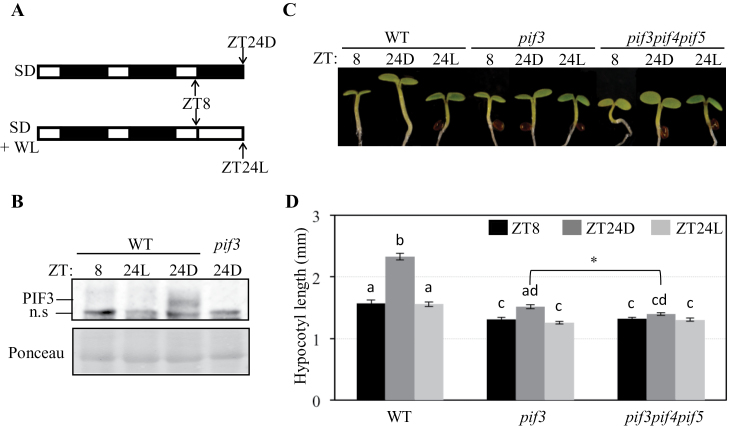
Exposure to WL prevents PIF3 accumulation and growth under SD conditions. (A) Diagram of the growth regime used for B–D. Seedlings were grown under SD conditions for 2 d and 8h, at which time (ZT8) they either were kept under SD and experienced a 16h night (ZT24D), or were moved to WL for the subsequent 16h (ZT24L). (B) Immunoblot of protein extracts from WT seedlings at the specified time points. A PIF3-specific polyclonal antibody was used as probe. As an antibody specificity control, a protein extract from *pif3-3* harvested at ZT24D was included. Ponceau staining was used as a loading control (bottom). n.s., non-speciﬁc cross-reacting bands. (C) Visual phenotype of WT, *pif3*, and *pif3pif4pif5* seedlings grown as detailed in A. (D) Hypocotyl length in WT, *pif3*, and *pif3pif4pif5* seedlings grown as detailed in A. Data represent the mean and SE of at least 20 seedlings. Different letters denote statistically significant differences among means defined by Tukey-b’s multiple comparison test (*P* < 0.05). Comparison between *pif3* and *pif3pif4pif5* genotypes in short-day conditions (ZT24D) fell short of statistical significance under the stringent Tukey-b statistical test but showed a statistically significant difference (*P* <0.05) by Student’s *t*-test (indicated with an asterisk). (This figure is available in colour at *JXB* online.)

**Fig. 2.**
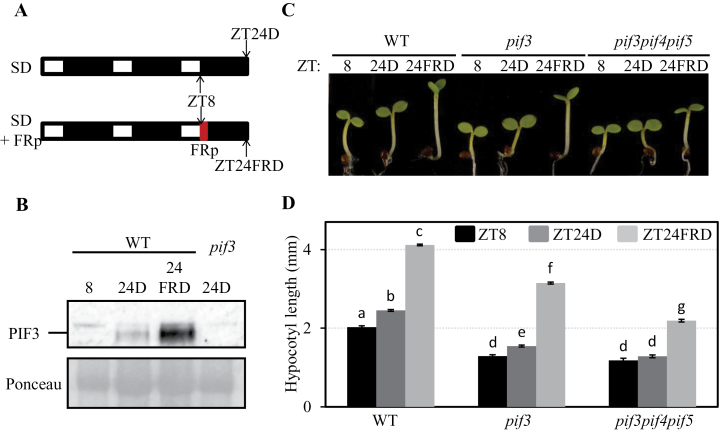
Inactivation of phytochrome activity by an EOD-FRp increases PIF3 accumulation and hypocotyl growth under SD conditions. (A) Diagram of the growth regime used for B–D. Seedlings were grown under SD conditions for 2 d and 8h, at which time (ZT8) they either were kept under SD and experienced a 16h night (ZT24D), or were given an EOD-FRp before the night period (ZT24FRD). (B) Immunoblot of protein extracts from WT seedlings at the specified time points. A PIF3-specific polyclonal antibody was used as probe. As an antibody specificity control, a protein extract from *pif3-3* harvested at ZT24D was included. Ponceau staining was used as a loading control (bottom). n.s., non-speciﬁc cross-reacting bands. (C) Visual phenotype of WT, *pif3*, and *pif3pif4pif5* seedlings grown as detailed in A. (D) Hypocotyl length in WT, *pif3*, and *pif3pif4pif5* seedlings grown as detailed in A. Data represent the mean and SE of at least 20 seedlings. Different letters denote significant differences among means (*P* < 0.05). (This figure is available in colour at *JXB* online.)

**Fig. 3.**
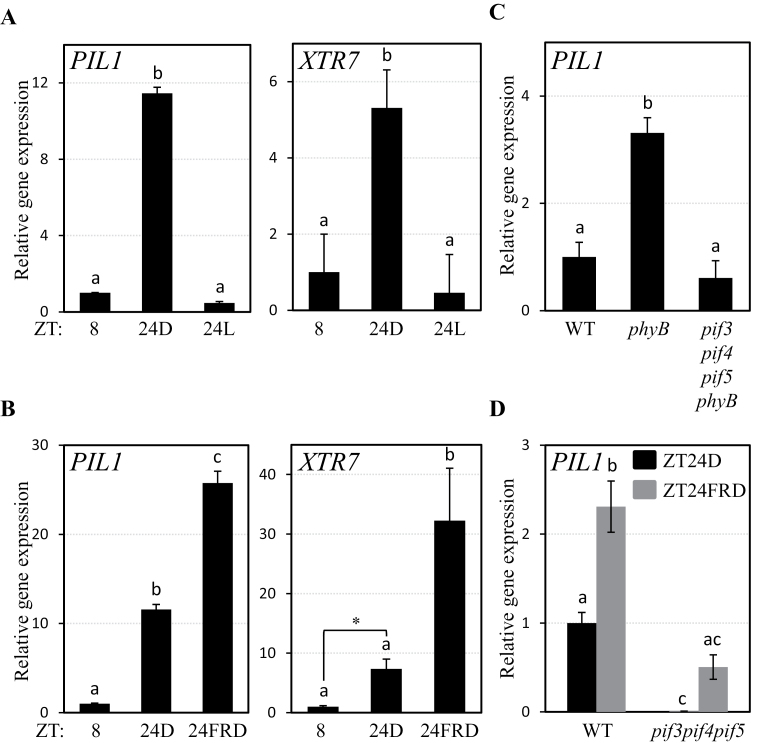
Correlation of growth marker gene expression with hypocotyl growth and levels of PIF3 under SD conditions. (A) Expression of *PIL1* and *XTR7* in seedlings grown as detailed in Fig. 1A. (B) Expression of *PIL1* and *XTR7* in samples grown as detailed in Fig. 2A. (C) Expression of *PIL1* in 3-day-old SD-grown WT, *phyB*, and *pif3pif4pif5phyB*. (D) Expression of *PIL1* in seedlings grown as detailed in Fig. 2A. The numerical value for *pif3pif4pif5* at ZT24D is <0.01. Expression of *PIL1* and *XTR7* was analysed by quantitative RT–PCR, and values were normalized to *PP2A*. Different letters denote significant differences among means (*P* < 0.05). Comparison between *XTR7* expression at ZT8 and ZT24D in B fell short of statistical significance under the stringent Tukey-b statistical test but showed a statistically significant difference (*P* < 0.05) by Student’s *t*-test (indicated with an asterisk).

**Fig. 4.**
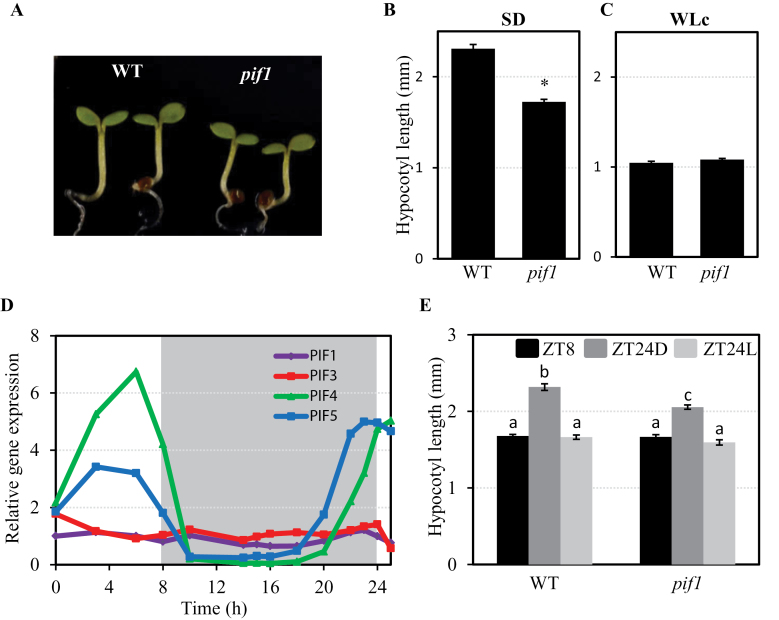
PIF1 promotes growth under SD conditions. (A) Visual phenotype of 3-day-old SD-grown WT and *pif1* seedlings. (B) Hypocotyl length in 3-day-old SD-grown WT and *pif1* seedlings. The asterisk indicates statistically significant differences (*P-*value <0.05). (C) Hypocotyl length in 3-day-old WT and *pif1* seedlings grown under continuous WL (WLc). (D) Expression of *PIF1*, *PIF3*, *PIF4*, and *PIF5* was analysed by quantitative RT–PCR. Values were normalized to *PP2A*, and expression levels relative to *PIF1* at 0h are shown. Data represent the mean of technical replicates. (E) Hypocotyl length of 2-day-old SD-grown WT and *pif1* grown as detailed in Fig. 1A. Data represent the mean and SE of at least 20 seedlings. Different letters denote significant differences among means (*P* < 0.05).

**Fig. 5.**
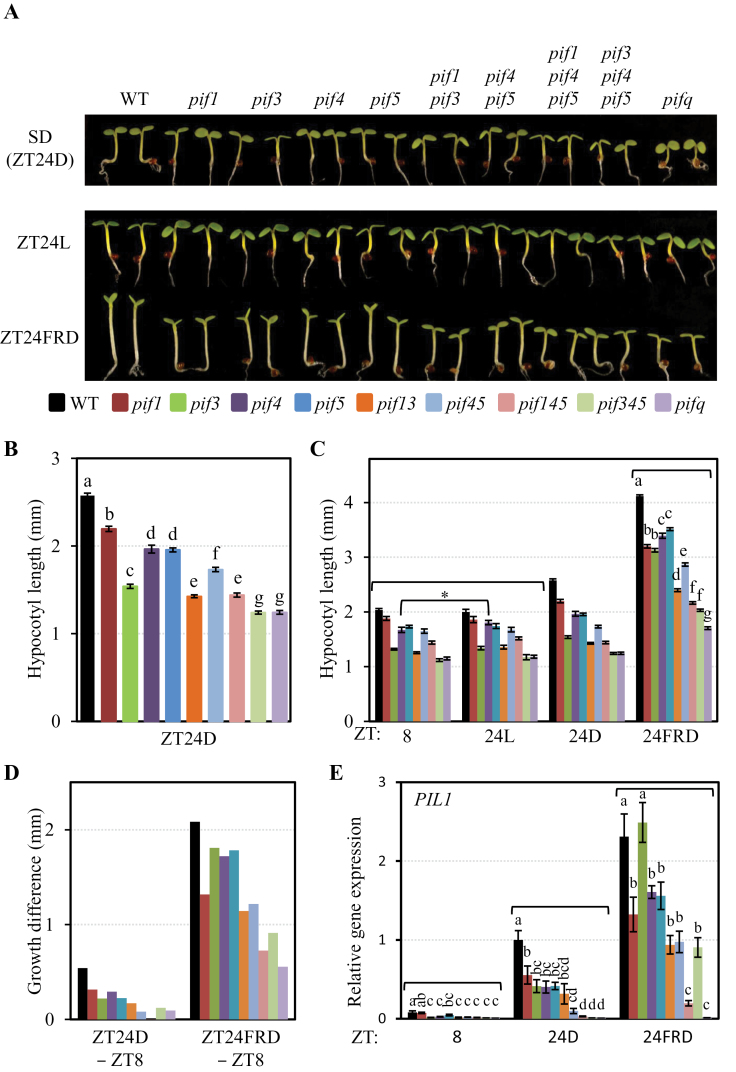
Morphological and gene expression analysis of *pif1* and higher order *pif* mutant combinations under SD conditions provides evidence for differential contributions of individual PIF quartet members. (A) Visible phenotypes of WT, *pif1*, *pif3*, *pif1pif3*, *pif4pif5*, *pif1pif4pif5*, *pif3pif4pif5*, and *pifq* mutant seedlings grown for 3 d in SD conditions (ZT24D) (top), or in ZT24L and ZT24FRD as specified in Fig. 1A and 2A, respectively (bottom). (B) Quantification of the hypocotyl elongation of 3-day-old SD-grown (ZT24D) WT and *pif*-mutant seedlings shown in A (top). Data represent the mean and SE of at least 20 seedlings. Different letters indicate signiﬁcant differences among means (*P*-value <0.05). (C) Quantification of the hypocotyl elongation of 3-day-old ZT24FRD- and ZT24L-grown WT and *pif*-mutant seedlings shown in A (bottom), compared with SD-grown (ZT24D) seedlings and seedlings grown under SD conditions for 2 d and 8h (ZT8). Data represent the mean and SE of at least 20 seedlings. For ZT24FRD, different letters denote significant differences among means (*P* < 0.05). In the case of ZT8 and ZT24L, the genotypes that show a statistical difference in ZT24L compared with ZT8 are indicated with an asterisk. (D) Differential growth responsiveness during the night period was calculated from data shown in C. Mean hypocotyl length at ZT8 was subtracted from the mean hypocotyl length at ZT24D or ZT24FRD for each genotype. The growth difference of *pif1pif4pif5* in ZT24D was nearly zero. (E) *PIL1* gene expression in WT, and *pif1*, *pif3*, *pif1pif3*, *pif4*, *pif5*, *pif4pif5*, *pif3pif4pif5*, and *pifq* mutant seedlings grown under SD conditions for 2 d and 8h (ZT8), 3 d (ZT24D), or under ZT24FRD conditions as specified in Fig. 2A. Different letters denote significant differences among means (*P* < 0.05). Most mutant combinations at ZT8, *pif1pif4pif5*, *pif3pif4pif5*, and *pifq* at ZT24D, and *pifq* at ZT24FRD had expression levels near zero.

For quantitative reverse transcription–PCR (RT–PCR) analysis, RNA extraction, cDNA synthesis, and qRT–PCR were done as described (Sentandreu *et al.*, 2011). Gene expression data in Figs 1, 2, 3, 4E, and 5 represent the mean of three biological replicates (each one measured in three technical replicates), and bars represent the SE. Gene expression data in detailed kinetics in Fig. 4D represent the mean of three technical replicates of one representative biological replicate. *PP2A* (*AT1G13320*) was used as a normalization control as described (Shin *et al.*, 2007). Gene expression analysis of *PIF3*, *PIF4*, *PIF5*, *XTR7*, and *PIL1* was done using the primers described in Soy *et al.* (2012). For *PIF1*, 5′-ATCCAACCTCGGGCCAGCCT-3′ and 5′-TTGGGTCGGGTGGAGACCGC-3′ were used as forward and reverse primers, respectively.

### Statistics

Gene expression and hypocotyl length data shown in Figs 1D, 2D, 3 A–D, 4E, 5B, C, and E were analysed by one-way analysis of variance (ANOVA) and the differences between means were evaluated by using Tukey-b post-hoc multiple comparison test (IBM SPSS Statistics Software). Data shown in Fig. 4B and C were submitted to a Student’s *t*-test analysis, as well as comparison between *pif3* and *pif3pif4pif5* ZT24D samples in Fig. 1D, and *XTR7* expression between ZT8 and ZT24D in Fig. 3B to complement the analysis by Tukey-b. In all cases, statistically significant differences were defined as those with a *P-*value <0.05.

## Results

### Exposure of SD-grown seedlings to constant light prevents PIF3 accumulation and leads to growth arrest

It has previously been described that phytochrome-imposed oscillations in PIF3 protein abundance regulate hypocotyl growth under SD conditions (Soy *et al.*, 2012). Under this photoperiodic growth regime, PIF3 levels stay low during the day and progressively accumulate during the dark hours to peak at the end of the night, coinciding with the maximum growth rate. Based on these results, it was hypothesized that alterations in PIF3 protein accumulation during the night period would have an impact on growth under SD conditions. To test this possibility, wild-type (WT) seedlings were first grown under SD conditions for 2 d, and then transferred to WL at the end of the third day (ZT8) for 16h (Fig. 1A). Controls were kept under SD conditions and experienced a subsequent 16h long night (Fig. 1A). The accumulation of endogenous PIF3 under these two conditions was then examined. PIF3 was below the detection level at ZT8, but was clearly detectable after 16h of darkness (ZT24D) (Fig. 1B) in accordance with previously reported data (Soy *et al.*, 2012). In contrast, PIF3 levels in seedlings transferred to WL remained below the detection level (ZT24L) (Fig. 1B). These results suggest that, in SD-grown seedlings, the night period is necessary to allow for accumulation of PIF3.

Next seedling growth in these conditions was monitored by comparing the hypocotyl length of SD-grown seedlings at ZT8 with the length of seedlings that were subsequently exposed to 16h darkness (ZT24D) or WL (ZT24L). As shown in Fig. 1C and D, 2-day-old SD-grown WT seedlings experienced significant hypocotyl elongation during exposure to the third night (between ZT8 and ZT24D), in accordance with previous reports (Nozue *et al.*, 2007; Soy *et al.*, 2012). In contrast, WT seedlings kept under WL did not exhibit any significant hypocotyl growth during the same 16h period (compare ZT8 with ZT24L). PIF3-deficient seedlings were shorter at ZT8 compared with the WT, and growth activity in the dark between ZT8 and ZT24D was also significantly reduced compared with the WT (Fig. 1C, D), in accordance with previous data (Soy *et al.*, 2012). Growth activity was also below detection when *pif3* seedlings were transferred to WL (compare ZT8 with ZT24L) (Fig. 1C, D). Together, these results support the notion that, under SD conditions, PIF3 accumulation during the night is necessary to induce growth, and substitution of the dark period by WL prevents PIF3 accumulation and leads to growth arrest.

PIF4 and PIF5 are positive regulators of growth under SD conditions together with PIF3 (Nozue *et al.*, 2007; Niwa *et al.*, 2009; Soy *et al.*, 2012). In accordance with this, *pif3pif4pif5* seedlings were slightly shorter at ZT24D compared with *pif3* (Fig 1D, and also see below Figs 2D and 5B), whereas exposure of *pif3pif4pif5* seedlings to 16h of WL instead of darkness did not lead to detectable growth, as observed for *pif3* (compare ZT24L with ZT8) (Fig. 1C, D). Together with previous data showing that accumulation of PIF4 and PIF5 under SD conditions occurs in the dark (Nozue *et al.*, 2007; Yamashino *et al.*, 2013), these data suggest that, under SD conditions, PIF4 and PIF5 accumulation during the night is also necessary to induce growth.

### Inactivation of phytochrome activity by an EOD-FRp increases PIF3 accumulation and leads to enhanced growth under SD conditions

To examine further how alterations in PIF3 protein accumulation during the night period have an impact on growth under SD conditions, and based on previous results showing that active Pfr phyB operates during the first hours of the night to prevent accumulation of PIF3 (Monte *et al.*, 2004; Soy *et al.*, 2012), seedlings were next treated with a saturating 15min FRp at the end of the third day (ZT8 time point) (Fig. 2A). It was expected that this EOD-FRp would lead to an increase in PIF3 levels during the night by rapidly inactivating phyB. Indeed, compared with control seedlings under SD conditions (ZT24D) (Fig. 2A), it was observed that PIF3 levels were increased by at least 2-fold in seedlings exposed to SD conditions supplemented by an EOD-FRp (ZT24FRD) (Fig. 2B).

Next the effect of the EOD-FRp on growth was examined by comparing the hypocotyl length at ZT8 with that at ZT24FRD. Control seedlings (ZT24D) were exposed to 16h of dark after ZT8. As shown in Fig. 2C and D, WT seedlings exposed to a 15min EOD-FRp and then kept in the dark for 16h exhibited an increase in hypocotyl length (from 2mm to 4mm) (compare ZT24FRD with ZT8) that was much more pronounced than the elongation observed during the same time period in WT control seedlings kept under SD conditions without an EOD-FRp (from 2mm to 2.4mm) (compare ZT24D with ZT8). Together, these data show a strong correlation between the increase in PIF levels during the night hours and the increase in hypocotyl growth during the same long-night period, and suggest that, under SD conditions, inactivation of phyB by an EOD-FRp leads to an increase in PIF3 accumulation during the night (and possibly other PIFs such as PIF4 and PIF5) that accelerates growth.

To determine whether and to what extent PIF3 and/or other PIF factors mediate this accelerated growth in response to an EOD-FRp, the effect of an EOD-FRp was examined in *pif3* and *pif3pif4pif5* mutants. Figure 2C and D shows that *pif3* mutants exhibited a robust growth response to EOD-FRp that was only slightly reduced in magnitude compared with that displayed by the WT (from 1.3mm to 3.1mm) (compare ZT24FRD with ZT8). In contrast, *pif3pif4pif5* seedlings had a significantly reduced hypocotyl response to the EOD-FRp compared with the WT or *pif3* (from 1.2mm to 2.2mm) (Fig. 2C, D). Together, these results suggest that an EOD-FRp triggers an increase in PIF3 protein accumulation during the night (as shown in Fig. 2B) and probably also in PIF4 and/or PIF5, and support the notion that the PIFs function redundantly to mediate phytochrome-regulated growth under SD conditions. Interestingly, triple *pif3pif4pif5* mutants still exhibited a significant elongation in response to EOD-FRp (Fig. 2C, D), indicating that at least one additional factor participates in the phytochrome-regulated growth response under these conditions.

### Expression of growth marker genes correlates with hypocotyl growth and with levels of PIF3 under SD conditions

Previously it was shown that the phytochrome-regulated growth-marker genes *PIL1* and *XTR7* (Salter *et al.*, 2003; Lorrain *et al.*, 2008; Leivar *et al.*, 2009; Nozue *et al.*, 2011) are direct targets of PIF3 under SD conditions, and are specifically induced at the end of the night with an expression peak that coincides with the moment of maximum growth (Soy *et al.*, 2012). To test whether the expression of these genes is affected under SD conditions when the levels of PIF3 (and probably other PIFs) are altered (see above, Figs 1, 2), the expression of *PIL1* and *XTR7* was analysed in 2-day-old SD-grown seedlings exposed to WL or to an EOD-FRp during the third day of growth (following the light protocols shown in Figs 1A and 2A). As presented in Fig. 3A, the expression levels of *PIL1* and *XTR7* were induced at the end of the night in response to 16h of darkness (ZT24D) compared with levels at the beginning of the night (ZT8 time point), in accordance with published data (Soy *et al.*, 2012). However, when seedlings were instead kept in WL during the same period of time (ZT24L), the expression levels of *PIL1* and *XTR7* were similar to the levels at the beginning of the night (ZT8) (Fig. 3A). Together with the PIF3 protein data shown in Fig. 1B, these results suggest that PIF3 accumulation during the night is necessary to induce expression of target genes such as *PIL1* and *XTR7*. Next the expression of *PIL1* and *XTR7* was examined 24h after an EOD-FRp (ZT24FRD) (Figs 2A, 3B). In these conditions, *PIL1* and *XTR7* expression is induced with respect to ZT8, and to levels ~3-fold higher compared with the controls without an EOD-FRp (ZT24D) (Fig. 3B). Together with the higher PIF3 protein accumulation shown in Fig. 2B, these results suggest that an increase in PIF3 protein accumulation following an EOD-FRp during the night (and possibly other PIFs such as PIF4 and PIF5) leads to enhanced expression of target genes such as *PIL1* and *XTR7*.

An antagonistic functional relationship in the regulation of hypocotyl growth under SD conditions has been described between PIF3, PIF4, PIF5, and phyB (Niwa *et al.*, 2009; Soy *et al.*, 2012). In contrast to the short hypocotyl of *pif3*, *pif4pif5*, and *pif3pif4pif5*, *phyB* mutant seedlings exhibit more elongated hypocotyls than the WT. The *phyB* tall phenotype was partially suppressed by genetic removal of PIF3, and further suppressed by additional genetic removal of PIF4 and PIF5, suggesting that higher PIF protein accumulation during the night in the absence of phyB is necessary for full expression of the *phyB* phenotype (Soy *et al.*, 2012). To examine whether the described correlation between PIF levels and *phyB* hypocotyl elongation was also observed at the gene expression level, gene expression analysis was performed in 3-day-old SD-grown *phyB* and WT seedlings at ZT24D. The expression level of *PIL1* was increased by >3-fold in *phyB* compared with the WT (Fig. 3C), an increase that was similar in magnitude to that observed in the WT after an EOD-FRp compared with WT seedlings kept in SD conditions without an EOD-FRp (compare ZTD and ZTFRD time points in Fig. 3B). In addition, this *PIL1* increase was suppressed in *pif3pif4pif5phyB* mutants (Fig. 3C). Together, these data indicate that the increased accumulation of PIF3, PIF4, and PIF5 in the absence of photoactive phyB (when removed either genetically or by an EOD-FRp) induces overexpression of *PIL1*, and suggest a correlation between elevated levels of PIF proteins, increased expression levels of growth-related genes, and the elongated hypocotyl of *phyB*.

To examine further the role of the PIFs in inducing growth-related gene expression, *PIL1* induction was next examined in 3-day-old SD-grown triple *pif3pif4pif5* mutants in response to an EOD-FRp (Fig. 3D). A significantly reduced response in comparison with the WT was detected, confirming the role of PIF3, PIF4, and PIF5 as positive regulators of *PIL1* expression after an EOD-FRp. Interestingly, although reduced in magnitude with respect to the WT, *pif3pif4pif5* mutant seedlings still responded to an EOD-FRp for *PIL1* expression (compare ZT24FRD with ZT24D), suggesting that at least one additional factor participates in the phytochrome-regulated gene induction response under these conditions.

### PIF1 regulates hypocotyl growth under SD conditions

The observation that *pif3pif4pif5* seedlings exhibit a reduced but still significant growth response and expression of growth marker genes after an EOD-FRp (Figs 2D, 3D) suggests that factors other than PIF3, PIF4, and PIF5 might be involved in the regulation of hypocotyl growth at night. This prompted the testing of whether PIF1 could be participating in this response under SD conditions. Hypocotyls of 3-day-old SD-grown *pif1* mutant seedlings were ~20% shorter compared with the WT (Fig. 4A, B). In comparison with SD conditions, WT seedlings exhibited shorter hypocotyls when grown under continuous WL for 3 d (WLc) (Fig. 4B, C), and WLc-grown *pif1* mutants were not significantly shorter than the WT (Fig. 4C). These data suggest that PIF1 is a component of the cellular machinery that induces growth during the night hours in SD conditions.

To establish the pattern of *PIF1* expression under diurnal SD conditions, *PIF1* transcript levels were analysed over 24h during the third day of seedling growth under SD conditions and compared with the expression patterns of *PIF3*, *PIF4*, and *PIF5*. PIF1 levels remained fairly constant over the 24h photoperiod (Fig. 4D), similarly to the previously reported *PIF3* expression pattern under SD conditions, and in contrast to the oscillating levels of *PIF4* and *PIF5* (Fig. 4D) (Yamashino *et al.*, 2003; Nozue *et al.*, 2007; Soy *et al.*, 2012). This pattern of expression indicates that *PIF1* is not regulated by the circadian clock under SD conditions, in contrast to *PIF4* and *PIF5*, and instead suggests that PIF1 protein abundance is probably regulated post-transcriptionally by the phytochromes as described for PIF3 (Soy *et al.*, 2012). Accordingly, phytochrome-imposed post-transcriptional regulation would keep PIF1 levels in SD-grown seedlings very low during the light hours, and would allow progressive accumulation during the night. This possibility is in accordance with previous data showing that transfer to the dark induced re-accumulation of LUC activity in LUC–PIF1-overexpressing seedlings under day-neutral conditions (Shen *et al.*, 2005). In agreement with this, a contribution of PIF1 to growth was detected during the 16h dark period in SD-grown seedlings, with *pif1* seedlings displaying reduced hypocotyl growth compared with the WT (ZT24D, Fig. 4E), but not when seedlings were kept in WL (ZT24L, Fig. 4E). These results are similar to those for PIF3 (Fig. 1), and support the notion that the night period is necessary for PIF1 accumulation.

### Phenotypic analysis of *pif* mutant combinations provides evidence for overlapping and differential contributions of individual PIFs to growth under SD conditions

To obtain insight into the contribution of PIF1 to the promotion of hypocotyl elongation under SD conditions relative to that of PIF3, PIF4, and PIF5, the hypocotyl length of 3-day-old SD-grown *pif1*, *pif3*, *pif4*, *pif5*, *pif1pif3*, *pif4pif5*, *pif1pif4pif5*, *pif3pif4pif5*, and *pif1pif3pif4pif5* (*pifq*) mutant seedlings was first analysed (Fig. 5A top, B). Under these conditions, *pif1* showed a significantly shorter hypocotyl than the WT (as also shown in Fig. 4), whereas *pif4* and *pif5* were similar and shorter than *pif1*, and *pif3* displayed the strongest phenotype of all four single mutants. Double *pif4pif5* mutants showed a short-hypocotyl phenotype similar to *pif3*, although slightly less robust (Fig. 5A top, B). Genetic removal of PIF1 in *pif3* and *pif4pif5* resulted in marginally shorter hypocotyls in both *pif1pif3* and *pif1pif4pif5* mutants (Fig. 5A top, B). Moreover, triple *pif3pif4pif5* seedlings had shorter hypocotyls than *pif1pif3* or *pif1pif4pif5*, and were similar to *pifq* (Fig. 5A top, B). Together, these results suggest that PIF1, PIF3, PIF4, and PIF5 collectively function in the promotion of growth under SD conditions, with the role of PIF3 probably being more prominent and similar to that of PIF4 and PIF5 combined, and with PIF1 contributing to a lesser extent.

As shown above, the 16h night period is necessary to induce growth under SD conditions, as WT seedlings arrested their hypocotyl growth when they were transferred to 16h of WL during the night h (Figs 1, 4), whereas an EOD-FRp given before the dark period accelerated WT growth during the subsequent 16h of darkness (Fig. 2). Comparison of the hypocotyl elongation at ZT8 and ZT24L in the WT and *pif* mutant seedlings examined in Fig. 5B (see Fig. 1A for a description of the experimental design) showed that WL treatment arrested seedling growth in all genotypes as expected (except for *pif4*, where growth was statistically significant although marginal) (compare ZT24L with ZT8, Fig. 5C), whereas an EOD-FRp (see Fig. 2A for a description of the experimental design) induced hypocotyl elongation to various degrees depending on the genotype (compare ZT24FRD with ZT8, Fig. 5C). To determine the contribution of PIF1 to the regulation of growth following an EOD-FRp-SD, and the possible interaction of PIF1 with the other PIF members under these conditions, the hypocotyl length of the various *pif* mutant combinations was analysed after the EOD-FRp treatment (ZT24FRD) (Fig. 5A bottom, C). Under these conditions, *pif* mutant seedlings displayed attenuated responses of different magnitude with respect to the WT (Fig. 5A bottom, C). All *pif* single mutants showed short hypocotyls compared with the WT, and this attenuated response to EOD-FRp was further reduced in the *pif1pif3* and *pif4pif5* double mutants, and even more in the triple *pif1pif4pif5* and *pif3pif4pif5* mutants (Fig. 5A bottom, C). These results suggest that all PIFs contribute to the promotion of growth in response to an EOD-FRp under SD conditions. In addition, given that the *pif1* mutant shows a phenotype similar to the other *pif* single mutants at ZT24FRD, and that the hypocotyl phenotype of *pif1* at ZT8 and ZT24D compared with the WT is only modest compared with the other *pif* single mutants, these data suggest that the relative contribution of PIF1 might be quantitatively more important after an EOD-FRp compared with its contribution under regular SD conditions (Fig. 5B, C). Indeed, growth difference measurements between ZT24FRD and ZT8 to quantify the elongation growth experienced during the 16h night after the EOD-FRp indicate that the *pif1* single mutant has a more attenuated response in comparison with *pif3*, *pif4*, and *pif5* (Fig. 5D). These results thus suggest that PIF1 might have a more prominent relative contribution to growth compared with PIF3, PIF4, and PIF5 after an EOD-FRp, compared with under SD conditions (Fig. 5B–D).

### PIF1 regulates expression of the growth-related *PIL1* gene under SD conditions, together with PIF3, PIF4, and PIF5

The observed contribution of PIF1 to seedling growth in SDs (Figs 4, 5 A–D) suggests that PIF1 might also contribute to the promotion of expression of growth-related genes targeted by PIF3, PIF4, and PIF5 under these conditions, such as *PIL1* (Soy *et al.*, 2012). Expression analyses in 3-day-old SD-grown seedlings (ZT24D) indicated that the promotion of *PIL1* transcript levels observed in the WT during the night hours is reduced in *pif1* similarly to *pif3*, *pif4*, and *pif5*, whereas *PIL1* levels in *pif1pif4pif5*, *pif3pif4pif5*, and *pifq* at ZT24D were all below the level of detection, indicating possible additive effects of the contribution of PIFs in higher order mutants (Fig. 5E). Together, this expression pattern supports the conclusion that PIF1 contributes to growth under diurnal conditions by promoting the expression of growth-related genes together with PIF3, PIF4, and PIF5.

Next the role of PIF1 in promoting gene expression in response to an EOD-FRp under SD conditions was examined. Compared with *PIL1* expression levels in WT seedlings, the expression in *pif1* was significantly reduced (Fig. 5E), and this effect was more robust compared with that in *pif3* (which showed no difference compared with the WT), and similar to that of *pif4* and *pif5*, *pif4pif5*, and *pif1pif3* double mutants, and *pif3pif4pif5* (Fig. 5E). Significantly, expression levels in *pif1pif4pif5* were greatly reduced compared with *pif4pif5*, and removal of PIF1 from *pif3pif4pif5* in the *pifq* mutant resulted in *PIL1* levels below detection (Fig. 5E). Together, this expression pattern is broadly consistent with the morphological phenotypes of the various *pif* mutant combinations after an EOD-FRp presented in Fig. 5A–D (although PIF3 seemed to have a less important role in the regulation of *PIL1* expression compared with its contribution to hypocotyl growth), and supports the conclusion that PIF1, PIF3, PIF4, and PIF5 collectively contribute to growth after an EOD-FRp by promoting the expression of growth-related genes, with PIF1 having a relatively more important role in these conditions compared with under SD conditions.

## Discussion

Previously the role of PIF3 as a prominent promoter of rhythmic growth under diurnal conditions together with PIF4 and PIF5 was defined, through direct regulation of growth-related genes at dawn coinciding with a PIF3 accumulation peak generated by phytochrome-imposed oscillations in protein abundance (Soy *et al.*, 2012). The experiments presented here examine the correlation under diurnal conditions between the levels of PIF3 during the night and the promotion of growth, by comparing PIF3 accumulation and hypocotyl elongation in SD conditions, and SD-grown seedlings released into WL or exposed to an EOD-FRp for 1 d. The data indicate a direct correlation between phytochrome activity during the night period, PIF3 levels (and possibly levels of other PIFs), and the extent of the growth response, and suggest that it occurs at least in part through the regulation of growth-related gene expression. In addition, combination of EOD-FRp and SD experiments uncovered PIF1 as a novel contributor to growth under light–dark conditions. Moreover, morphogenic and marker gene expression evidence is provided that individual members of the PIF quartet (PIF1, PIF3, PIF4, and PIF5) contribute differentially to the promotion of seedling growth, suggesting that they act together with partially redundant functions to optimize growth under diurnal conditions.

The observation that substitution of the 16h dark period by WL led to seedling growth arrest under SD conditions provides evidence that night-induced inactivation of phytochromes and subsequent accumulation of the PIFs are necessary to promote growth (Fig. 1), although additional involvement of other photoreceptors such as cryptochromes, which have been previously shown to participate in the control of photoperiodic growth (Mazzella and Casal, 2001), cannot be discarded. Further support for a direct correlation between PIF levels and the magnitude of the growth response was observed when giving an EOD-FRp before the beginning of the 16h night period. This treatment promoted overaccumulation of PIF3 and possibly other PIF proteins, increased the expression of PIF-regulated growth-related genes, and enhanced hypocotyl growth by 3-fold during the night period (Figs 2, 3). Based on previous results (Soy *et al.*, 2012), it was expected that this EOD-FRp acted primarily through inactivation of the phytochrome system (mainly of phyB) at the start of the dark period. In agreement, *phyB* mutant seedlings grown under SD conditions, which display a tall phenotype and accumulate higher amounts of PIF3 during the night (Niwa *et al.*, 2009; Soy *et al.*, 2012), had increased expression of growth-related genes that were suppressed by genetic removal of PIF3, PIF4, and PIF5 (Fig. 3). These data thus provide additional support for a strong correlation between increased PIF levels during the night hours under SD conditions and enhanced hypocotyl growth, and are in agreement with previous data in seedlings exposed to FR light-enriched environments such as vegetational shade, low R/FR ratios, or an EOD-FRp, where inactivation of the phytochromes triggers an increase in PIF abundance and a promotion of growth (Lorrain *et al.*, 2008; Leivar *et al.*, 2012a, b; Sellaro *et al.*, 2012).

The results presented here revealed that factors other than PIF3, PIF4, and PIF5 participate in the promotion of phytochrome-regulated growth under diurnal conditions, because the *pif3pif4pif5* triple mutant still responded both morphologically and molecularly to an EOD-FRp treatment given at the beginning of the night in SD conditions (Figs 2, 3), consistent with previous results in shade conditions (Leivar *et al.*, 2012a). The present phenotypic and marker gene expression analyses of *pif1* single and higher order mutants identify PIF1 as an additional factor that contributes to the promotion of growth under SD conditions together with PIF3, PIF4, and PIF5, albeit to a lesser extent, possibly by direct regulation of growth-related genes such as *PIL1* (Figs 4–6). Analyses of *pif1pif4pif5* and *pif3pif4pif5* hypocotyl length compared with *pifq* indicated that PIF3 alone was able partially to complement the *pifq* phenotype, whereas PIF1 was not, suggesting that PIF1 is required but not sufficient to promote growth in SD conditions in the absence of the other three PIFs, although a significant additive effect was observed when PIF1 was removed from *pif3* or *pif4pif5* mutants (Fig. 5). Examination of marker gene expression revealed a picture where the four PIFs collectively induce the expression of the growth marker gene *PIL1* (Fig. 5).

Interestingly, in contrast to SD conditions, PIF1 appears to have a more robust contribution to the promotion of hypocotyl elongation after an EOD-FRp, whereas PIF3, PIF4, and PIF5 contribute to a lesser extent (Fig. 5D). Under these conditions, the role of PIF1 was similar to the combined action of PIF4 and PIF5 (Fig. 5D). Analyses of *pif3pif4pif5* hypocotyl length compared with *pifq* indicated that PIF1 was able partially to complement the *pifq* phenotype at ZT24FRD but not at ZT24D (Fig. 5C), in agreement with the notion that PIF1 has a more predominant role after an EOD-FRp compared with SD conditions. Intriguingly, the *pifq* mutant still retained some ability to grow after an EOD-FRp (Fig. 5), suggesting that additional factors might contribute to the regulation of growth under SD conditions as previously described in shade (Leivar *et al.*, 2012b), and consistent with the possible participation of additional PIFs such as PIF7 (Leivar *et al.*, 2008a; Li *et al.*, 2012; EM and PL, unpublished). Examination of marker gene expression revealed a picture for relative PIF contribution broadly similar to that for hypocotyl elongation, with the four PIFs collectively inducing the expression of *PIL1*, with a more predominant contribution of PIF1 compared with PIF3 (Fig. 5). The data presented here show that treatment of SD-grown seedlings with an EOD-FRp induced exaggerated hypocotyl elongation and a robust increase in growth marker genes such as *PIL1* and *XTR7* (Figs 2, 3), with PIF1 having a prominent contribution in regulating these responses (Fig. 5). These characteristics resemble those of etiolated seedlings (Leivar *et al.*, 2009; Shin *et al.*, 2009), and suggest that SD-grown green seedlings exposed to an EOD-FRp might experience a partial reversal to the etiolated state, similar to what has been previously suggested for shade-induced responses (Leivar *et al.*, 2012b). In agreement with this possibility, SD-grown WT seedlings exposed to an EOD-FRp displayed partially closed cotyledons typical of etiolated seedlings (Fig. 5A). This response was absent in SD conditions or in SD-grown seedlings transferred to WL, and was dependent on PIF activity (Fig. 5A). Overall, the data support the notion that an increase in PIF levels in SD conditions after an EOD-FRp induces a partial reversion to the etiolated state and favours a more important relative contribution of PIF1. This change in PIF relative contribution between SDs and SDs supplemented with an EOD-FRp might include a change in relative activity, abundance, and/or binding affinity for target genes. Additional experiments will be required to elucidate the mechanisms involved.

Taken together, the data presented here indicate that, under SD conditions, there is a strong correlation between PIF protein levels and the levels of marker gene expression and hypocotyl growth. The present work suggests that phytochrome-regulated abundance of PIF levels is a central regulatory pathway that determines the magnitude of growth under diurnal conditions, in good agreement with the previously described role for the PIFs during seedling etiolation or shade avoidance (Bae and Choi, 2008; Leivar *et al.*, 2008b, 2012b; Lorrain *et al.*, 2008). How PIFs implement these growth responses is an active area of research. Current evidence indicates that PIFs directly regulate a subset of genes enriched in transcription factors and in synthesis and responses to auxin during seedling de-etiolation and responses to shade (Hornitschek *et al*., 2012; Zhang *et al.*, 2013). Under modified SD conditions, the PIF4- and PIF5-regulated transcriptional network has been defined and also includes auxin-related genes (Nozue *et al.*, 2011), although the direct targets in these conditions have not yet been determined. Further experiments are required to define the transcriptional network targeted by the PIF quartet under diurnal conditions. Comparative analysis of the PIFq-regulated transcriptome in SD conditions with that in de-etiolation or shade will establish whether regulation of diurnal growth involves targeting of SD-specific genes, or whether, and to what extent, these different phytochrome/PIF-dependent responses are implemented through a shared transcriptional network that drives common downstream facets of morphogenesis such as hypocotyl growth.
